# Atrial pacing successfully suppressed drug-resistant ventricular fibrillation in a patient with early repolarization syndrome

**DOI:** 10.1016/j.hrcr.2022.04.009

**Published:** 2022-04-19

**Authors:** Shushi Nishiwaki, Satoshi Shizuta, Munekazu Tanaka, Akihiro Komasa, Hirohiko Kohjitani, Takeshi Kimura

**Affiliations:** Department of Cardiovascular Medicine, Graduate School of Medicine, Kyoto University, Kyoto, Japan

**Keywords:** Early repolarization syndrome, Implantable cardioverter-defibrillator, Atrial pacing, Ventricular fibrillation, Electrocardiogram

**Key Teaching Points**

• Early repolarization syndrome is a lethal disease causing polymorphic ventricular tachycardia and ventricular fibrillation.
• Atrial pacing with a rate of 75 bpm markedly reduced the amplitudes of the J-waves and suppressed drug-resistant ventricular fibrillation in a patient with early repolarization syndrome.
• Atrial pacing with a transvenous implantable cardioverter-defibrillator may be an attractive non-invasive treatment option for the prevention of ventricular fibrillation in patients with early repolarization syndrome.

## Introduction

Early repolarization (ER) is a common finding in electrocardiograms (ECGs) of healthy subjects and used to be recognized as benign. But recently, especially in inferior and/or lateral leads, ER has been identified as a marker of polymorphic ventricular tachycardia (VT) or ventricular fibrillation (VF) leading to sudden cardiac death (SCD), which was named as early repolarization syndrome (ERS).^1^ ER is defined as a slur or notch on the terminal part of the QRS complex with an elevation of the ST segment, which is called an abnormal J wave.^2^ Because of similar ECG findings and clinical features in terms of ST-segment elevation, a structurally normal heart, and VT/VF mostly during sleep, it has been proposed to combine ERS and Brugada syndrome (BrS) as J-wave syndromes (JWSs),^2^ although the magnitude and lead location of abnormal J waves differ between ERS and BrS. An implantable cardioverter-defibrillator (ICD) is the only established therapy to prevent SCD in JWSs. However, it becomes necessary to suppress VT/VF in patients with frequent ICD shocks. To date, several drugs including isoproterenol, quinidine, bepridil, and cilostazol have been reported to suppress VT/VF in patients with JWSs.^2^^,^^3^ Antibradycardia pacing may also be effective, because VT/VF occur mostly during sleep in JWSs.^2^ However, there are only a few case reports of BrS showing the efficacy of antibradycardia pacing,^2^^,^^4^ with no such reports for ERS. Here we report a case of ERS in which atrial pacing with ICD markedly reduced the amplitude of J waves and suppressed drug-resistant VF causing frequent ICD shocks.

## Case report

**Figure 1:**
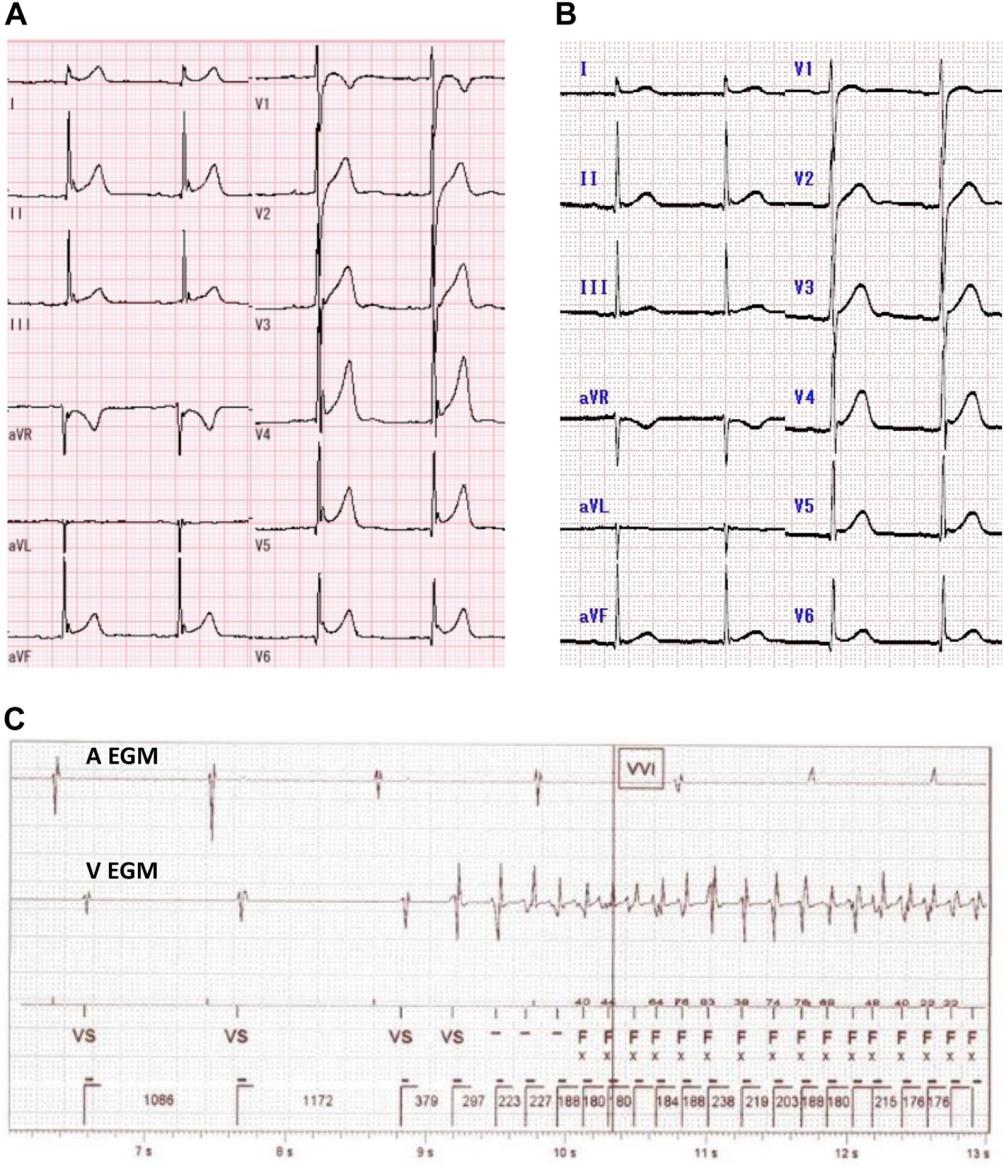
**A:** Electrocardiogram (ECG) at baseline (first hospitalization) without antiarrhythmic drug use. Distinct J waves were observed at inferior and lateral leads, with an average amplitude of 0.36 ± 0.09 mV. **B:** ECG at the time of hospital admission for transvenous implantable cardioverter-defibrillator battery exchange (second hospitalization) under quinidine 600 mg/day. The average amplitude of the J waves at inferior and lateral leads was 0.17 ± 0.05 mV, significantly lower than the baseline (*P* = .002). **C:** Intracardiac ECG of ventricular fibrillation (VF) preceded by sinus bradycardia on day 2 after battery exchange. The R-R interval just before the onset of VF was 1172 ms.

The patient was admitted to our hospital at 14 years of age because of a syncopal attack (first hospitalization). He had no family history of SCD. Transthoracic echocardiography showed normal findings. A 12-lead ECG demonstrated distinct abnormal J waves in inferior and lateral leads (Figure 1A). Although VT/VF was not induced in an electrophysiological study, spontaneous VF was recorded in the early morning, 12 hours after the study. Therefore, we diagnosed the underlying heart disease of VF as ERS and implanted a dual-chamber transvenous ICD (TV-ICD) (Ellipse DR 2277-36Q ICD; Abbott, Chicago, IL) for secondary prevention of SCD. Genetic examination showed no mutation in reported genes associated with ERS.

**Figure 2:**
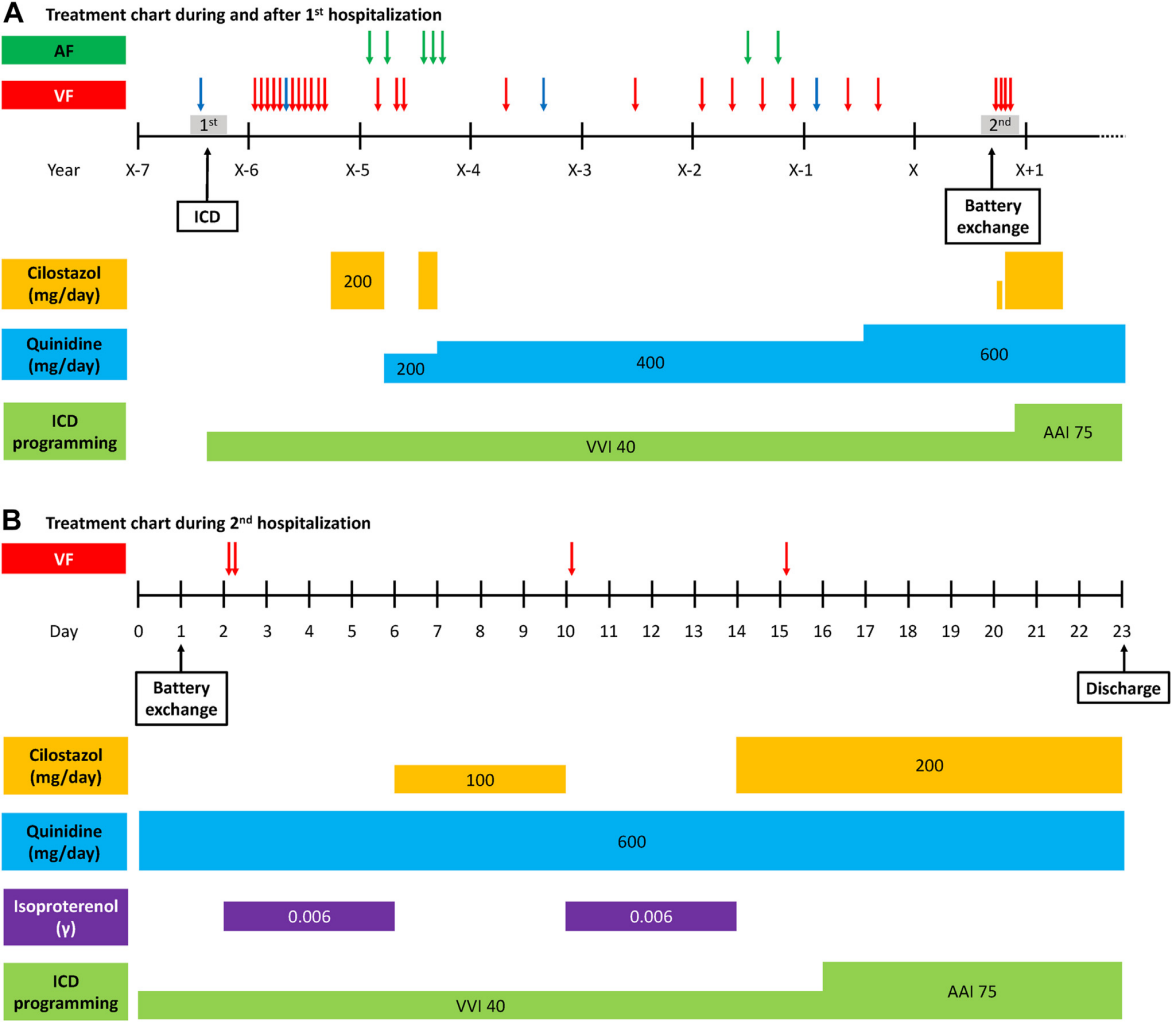
**A:** Treatment chart during the entire period, including first and second hospitalizations. **B:** Treatment chart during second hospitalization. Blue arrows indicate ventricular fibrillation attacks with spontaneous termination and red arrows indicate those with implantable cardioverter-defibrillator shocks. AF = atrial fibrillation; ICD = implantable cardioverter-defibrillator; VF = ventricular fibrillation.

At 1 year after TV-ICD implantation, cilostazol 200 mg/day was started because of multiple VF episodes leading to ICD shocks. Although most VF episodes with appropriate ICD shocks were during sleep, they were highly symptomatic. Cilostazol suppressed VF, but frequent symptomatic atrial fibrillation (AF) occurred. We switched cilostazol to quinidine 200 mg/day, but further VF attacks occurred. We next changed the pharmacological therapy to a combination of quinidine 200 mg/day and cilostazol 200 mg/day. However, because of recurrent symptomatic AF, cilostazol was discontinued and quinidine monotherapy was chosen, with a higher dose of 400 mg/day. Nevertheless, several VF episodes leading to ICD shocks were documented during the following 2 years. At 20 years of age, the patient’s quinidine dose was increased to 600 mg/day because of frequent VF attacks causing ICD shocks, along with 2 AF episodes. Only 1 VF episode was documented during the following 11 months, without AF (Figure 2A). Ten months after the last VF episode, he was admitted to our hospital for battery exchange of TV-ICD at 21 years of age (second hospitalization). An ECG at hospital admission showed reduced amplitudes of the J waves in inferior and lateral leads under 600 mg/day of quinidine (Figure 1B). The battery exchange (Ellipse DR 2377-36QC ICD; Abbott, Chicago, IL) was performed under mild conscious sedation with a low dose of midazolam. Although quinidine was not interrupted, 2 VF attacks followed by appropriate ICD shocks occurred in the early morning on day 2 (Figure 1C). At that time, he had a low-grade fever with a peak of 37.7°C. No electrolyte abnormalities were observed, including hypokalemia. The 12-lead ECG showed an apparent augmentation of the J waves in inferior and lateral leads, which suggested increased activity of ERS. Continuous intravenous isoproterenol decreased the amplitude of the J waves and suppressed VF. The low-grade fever resolved on day 3. On day 6, intravenous isoproterenol was discontinued and cilostazol 100 mg/day was started in addition to quinidine 600 mg/day. However, VF recurred on day 10, and isoproterenol was restarted. On day 14, cilostazol 200 mg/day was started and isoproterenol was discontinued. Nevertheless, VF recurred on day 15 (Figure 2B).

**Figure 3:**
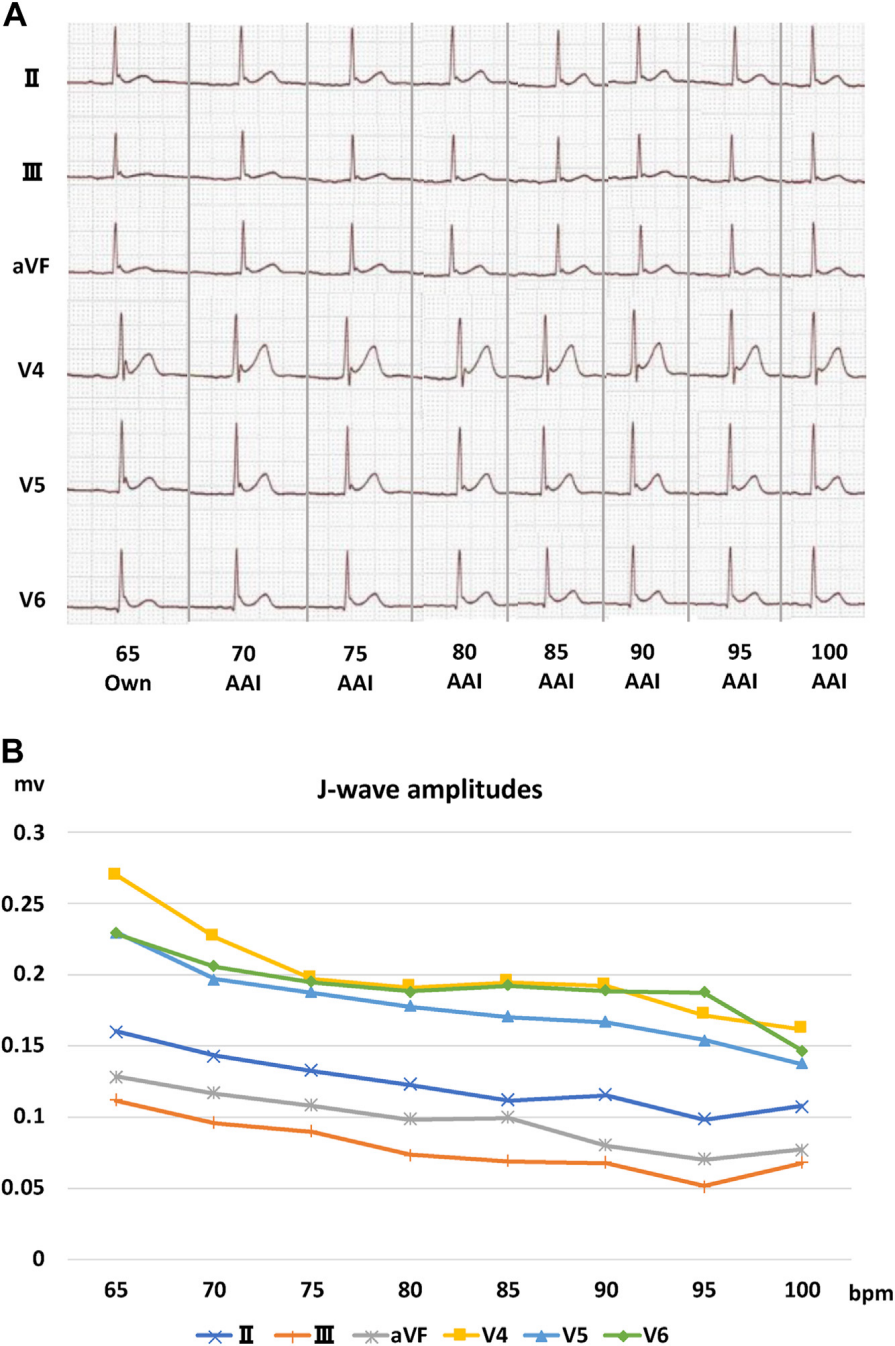
**A:** Changes of electrocardiograms at inferior and lateral leads at various heart rates. The J-wave amplitudes were markedly decreased by high-rate atrial pacing. **B:** Plots of the J-wave amplitudes at the inferior and lateral leads at various heart rates. The amplitudes of the J waves decreased according to heart rates, almost reaching a plateau at 75 beats/min. Abbreviations as in Figure 1.

Because most VF episodes had occurred in the early morning, with preceding heart rates of 50‒60/min (Supplemental Table 1), sinus bradycardia was considered an important trigger of VF. We therefore evaluated the effect of atrial pacing ranging from 70 to 100 beats per minute (bpm), using TV-ICD on the amplitudes of the J waves. A distinct inverse relation was observed between the heart rates and the J-wave amplitudes (Figure 3A). Because the J-wave amplitudes almost reached a plateau at 75 bpm (Figure 3B) and higher pacing rates were associated with substantial palpitation of the patient, the final pacing rate of TV-ICD was set at 75 bpm on day 16. The patient was discharged from hospital on day 23 with the combined medication of quinidine 600 mg/day and cilostazol 200 mg/day (Figure 2B). Thereafter, cilostazol was discontinued at 5 months. He has been free from VF for 14 months since discharge.

## Discussion

To the best of our knowledge, this is the first report showing the efficacy of atrial pacing in suppressing VF in a patient with ERS.

Although the mechanisms of VT/VF in ERS have not been well established, a repolarization hypothesis has been suggested.^2^ The transmural voltage gradient is increased in ERS patients, owing to an increased transient outward potassium current (I_to_) and decreased currents of inward channels of sodium and calcium (I_Na_ and I_Ca_),^5^ which develops phase 2 reentry, causing short-coupled premature ventricular beats leading to VT/VF.^5^

As a pharmacologic approach to suppressing VT/VF in ERS, several drugs are reported to be effective, including isoproterenol, quinidine, bepridil, and cilostazol.^2^ Because the increased I_to_ current is considered the most important mechanism of arrhythmogenesis in ERS, partial inhibition of this current is considered to be effective.^2^ Quinidine and bepridil reduce I_to_ directly, and isoproterenol and cilostazol increase I_Ca_ and thereby increase heart rate, leading to a reduction of I_to_.^2^ The combination of cilostazol and bepridil is also reported to be effective in suppressing VF in a patient with ERS.^6^ In the present case, the J-wave amplitudes under quinidine 600 mg/day before the ICD battery exchange were much lower than those at baseline without medications (Figure 1A and 1B), suggesting that quinidine was effective in suppressing the activity of ERS. However, the patient developed quinidine-resistant VF soon after the ICD battery exchange.

When the patient had 2 VF attacks on day 2 postsurgery, he had a low-grade fever with a peak of 37.7°C, which quickly resolved on day 3. Because previous reports have shown increased activity of JWSs under a febrile state,^2^^,^^7^ the VF attacks on day 2 may have been associated with the low-grade fever. However, VF attacks on day 10 and day 15 occurred under an afebrile state.

VF in ERS patients mostly occurs during sleep or at a low level of physical activity. This is because I_to_ current increases at slow heart rates.^2^ Because the recovery of I_to_ after inactivation is slow, atrial pacing with a relatively high rate is theoretically effective for ERS in suppressing VT/VF. Indeed, high-rate atrial pacing has been shown to reduce the amplitudes of J waves on body surface and epicardial ECGs in patients with ERS.^8^, ^9^, ^10^ However, only a few case reports of BrS have shown that continuous atrial pacing actually suppresses VF, and there is no such report for ERS.^4^ In the present case, atrial pacing at 75 bpm with TV-ICD markedly reduced the amplitudes of the J waves and, importantly, suppressed VF for more than a year. The pacing rate of 75 bpm was chosen because the amplitudes of the J waves almost reached a plateau at that rate, and atrial pacing at higher rates caused substantial palpitation of the patient at rest. In addition, atrial pacing at higher rates might be associated with Wenckebach-type atrioventricular block during sleep, diminishing the effect of the pacing. Thus, evaluation of the J-wave amplitudes as well as the patient’s symptoms at various rates of atrial pacing may be important when determining the appropriate pacing rate.

Because TV-ICD implantation is frequently associated with device-related complications such as device infection and lead fracture, subcutaneous ICD is reported to be a good alternative for JWSs.^11^^,^^12^ But for secondary-prevention patients, who are at higher risk of frequent VF attacks, a TV-ICD capable of atrial pacing may be desirable, rather than subcutaneous ICD.

## Conclusion

We reported an ERS case in which atrial pacing at 75 bpm with TV-ICD markedly reduced the amplitudes of J waves and suppressed drug-resistant VF for 14 months. The atrial pacing with TV-ICD may be an attractive noninvasive treatment option for the prevention of VF in ERS patients.
