# Supplementary material for: Atrial pacing successfully suppressed drug-resistant ventricular fibrillation in a patient with early repolarization syndrome
Source: HeartRhythm Case Rep. 2022 Apr 19;8(7):488–92. doi: 10.1016/j.hrcr.2022.04.009 (PMC9289062; doi:10.1016/j.hrcr.2022.04.009)
Supplement: Supplemental Table 1 [file mmc1.docx]

| **Supplemental Table. Characteristics of VF episodes** | | | | |
| --- | --- | --- | --- | --- |
| **VF episodes during and after first hospitalization and until second hospitalization** | | | | |
| **No** | **Time** | | **Heart rate (bpm)** | **Drug** |
| 1 | AM 5:05 | | 55 | None |
| 2 | AM 4:25 | | 58 | None |
| 3 | AM 5:15 | | 50 | None |
| 4 | AM 4:44 | | 48 | None |
| 5 | PM 0:44 | | 66 | None |
| 6 | AM 10:27 | | 56 | None |
| 7 | NA | | NA | None |
| 8 | NA | | NA | None |
| 9 | AM 3:07 | | 52 | None |
| 10 | AM 1:51 | | 67 | None |
| 11 | AM 2:53 | | 65 | None |
| 12 | AM 3:38 | | 73 | None |
| 13 | NA | | NA | None |
| 14 | NA | | NA | Cilostazol 200 mg |
| 15 | AM 4:04 | | 65 | Quinidine 200 mg |
| 16 | AM 4:33 | | 53 | Quinidine 200 mg |
| 17 | AM 3:47 | | 58 | Quinidine 400 mg |
| 18 | AM 4:41 | | 52 | Quinidine 400 mg |
| 19 | AM 4:27 | | 51 | Quinidine 400 mg |
| 20 | NA | | NA | Quinidine 400 mg |
| 21 | AM 8:42 | | 59 | Quinidine 400 mg |
| 22 | AM 11:14 | | 50 | Quinidine 400 mg |
| 23 | AM 4:10 | | 53 | Quinidine 400 mg |
| 24 | AM 2:07 | | 49 | Quinidine 400 mg |
| 25 | AM 10:13 | | 62 | Quinidine 400 mg |
| 26 | AM 7:13 | | 69 | Quinidine 600 mg |
| **VF episodes during second hospitalization** | | | | |
|  | **Day** | **Time** | **Heart rate (bpm)** | **Drug** |
| 1 | 2 | AM 6:35 | 51 | Quinidine 600 mg |
| 2 | 2 | AM 9:07 | 58 | Quinidine 600 mg |
| 3 | 10 | AM 6:17 | 58 | Quinidine 600 mg and cilostazol 100 mg |
| 4 | 15 | AM 8:40 | 66 | Quinidine 600 mg and cilostazol 200 mg |

NA, not available; VF, ventricular fibrillation; bpm, beats per minute.
